# Quantitative PCR Reveals Strong Spatial and Temporal Variation of the Wasting Disease Pathogen, *Labyrinthula zosterae* in Northern European Eelgrass (*Zostera marina*) Beds

**DOI:** 10.1371/journal.pone.0062169

**Published:** 2013-05-02

**Authors:** Anna-Christina Bockelmann, Verena Tams, Jana Ploog, Philipp R. Schubert, Thorsten B. H. Reusch

**Affiliations:** 1 Experimental Ecology – Food webs, Helmholtz Centre for Ocean Research (GEOMAR), Kiel, Germany; 2 Evolutionary Ecology of Marine Fishes, Helmholtz Centre for Ocean Research (GEOMAR), Kiel, Germany; Smithsonian's National Zoological Park, United States of America

## Abstract

Seagrass beds are the foundation species of functionally important coastal ecosystems worldwide. The world’s largest losses of the widespread seagrass *Zostera marina* (eelgrass) have been reported as a consequence of wasting disease, an infection with the endophytic protist *Labyrinthula zosterae*. During one of the most extended epidemics in the marine realm, ∼90% of East and Western Atlantic eelgrass beds died-off between 1932 and 1934. Today, small outbreaks continue to be reported, but the current extent of *L. zosterae* in European meadows is completely unknown. In this study we quantify the abundance and prevalence of the wasting disease pathogen among 19 *Z. marina* populations in northern European coastal waters, using quantitative PCR (QPCR) with primers targeting a species specific portion of the internally transcribed spacer (ITS1) of *L. zosterae*. Spatially, we found marked variation among sites with abundances varying between 0 and 126 cells mg^−1^ *Z. marina* dry weight (mean: 5.7 *L. zosterae* cells mg^−1^ *Z. marina* dry weight ±1.9 SE) and prevalences ranged from 0–88.9%. Temporarily, abundances varied between 0 and 271 cells mg^−1^ *Z. marina* dry weight (mean: 8.5±2.6 SE), while prevalences ranged from zero in winter and early spring to 96% in summer. Field concentrations accessed via bulk DNA extraction and subsequent QPCR correlated well with prevalence data estimated via isolation and cultivation from live plant tissue. *L. zosterae* was not only detectable in black lesions, a sign of *Labyrinthula*-induced necrosis, but also occurred in green, apparently healthy tissue. We conclude that *L. zosterae* infection is common (84% infected populations) in (northern) European eelgrass populations with highest abundances during the summer months. In the light of global climate change and increasing rate of marine diseases our data provide a baseline for further studies on the causes of pathogenic outbreaks of *L. zosterae.*

## Introduction

Seagrass beds are among the most threatened coastal ecosystems worldwide [1] while at the same time, they provide very important ecological functions as nursery habitat, sediment stabilizer, and via carbon and nutrient fixation [2]. We are now witnessing a century of accelerated seagrass decline driven by growing human populations, coastal development, ecological degradation and climate change [1], [3], [4]. However, the world’s largest and fastest losses of *Zostera marina* occurred in the 1930’s and were attributed to eelgrass wasting disease, caused by the net-slime mold *Labyrinthula zosterae* (Straminopiles, an endophytic protist reviewed by [5]). Among the many other known factors causing eelgrass decline, the role of pathogens has so far largely been neglected, although diseases are already noticeably on the increase not only in marine ecosystem [6], [7]. The main objective of this study was to obtain first quantitative data on the prevalence and abundance of the wasting disease pathogen *Labyrinthula zosterae* in contemporary *Z. marina* populations of Northern Europe.

Although detailed data are scarce, it is generally accepted that *Z. marina* beds were very common before the disease struck throughout the North Atlantic (see e.g. [8] for the Wadden Sea, [9] for the Netherlands, [10] for Danmark, [11] for the German Baltic and [12] 2008 for France). Historical records of a large eelgrass industry producing insulation and mattresses suggest high abundances of extended eelgrass beds in France, The Netherlands and Canada [13], [14]. This changed dramatically when in the 1930’s, a pandemic caused by the net-slime mold *L. zosterae* struck eelgrass beds on both sides of the North Atlantic. Beginning in 1930, eelgrass beds disappeared from large areas ranging from New Brunswick to north-west Carolina at the Atlantic West Coast within only two years [15], [16]. In 1931, similar die-offs were reported from Brittany and the Norman-Breton Gulf in France [17], and in the subsequent year from sublitoral eelgrass beds in the Dutch Wadden Sea [18]. In 1933, the epidemic reached southeast England [19], the northern German Wadden Sea [13] and the Danish west coast, while it arrived in Norway and the Baltic in 1934 [13], [20]. Eelgrass bed recolonization was slow and accompanied by new outbreaks until 1965 [12], [21]–[24]. In many regions, only intertidal meadows have recovered [8], [25], while subtidal *Z. marina* beds have never recovered and are today restricted to remnant patches within tidal creeks [26], [27]. In the 1980s new outbreaks of wasting disease were reported from the Atlantic coast of Nova Scotia and New England [24] and the Pacific northwest coast of North America [28], Brittany (France) and Grevelingen lagoon (The Netherlands, [24]), demonstrating that pathogenic strains of *L. zosterae* are still present in contemporary eelgrass beds.

During pathogenic outbreaks of *L. zosterae*, eelgrass plants exhibit a fast spread of black lesions on all leaves within hours, followed by leaf abscission, rhizome discoloration and mortality [29]–[31]. Even in the 1930’s, *Labyrinthula* was microscopically identified in diseased plants and experimental inoculation of healthy plants with infected leaves was reproducible [29]. In 1991, after the recurrence of wasting disease on the Atlantic and Pacific US coasts, Muehlstein et al. [30] identified *Labyrinthula zosterae* as the causative agent of wasting disease according to Kochs postulates. A recent survey of *Labyrinthula*-isolates (N = 53) from six northern European sites and one southern location (Adriatic Sea) identified three species based on a 1400 bp region of the 18S small subunit rDNA, all isolated from apparently healthy *Z. marina* beds. While the most common isolate was *L. zosterae*, two additional culturable *Labyrinthula* species were also found [32].

In order to quantify infection, Burdick et al. [33] introduced the “wasting index”, which estimates the percentage of necrotic tissue for each leaf on a vegetative shoot. Although valuable as a first step towards quantification, this indirect method has several disadvantages. First, not all lesions are caused by *Labyrinthula* spp. and second, not all *Labyrinthula* spp. result in observable lesions (see also [23]). Most importantly, we still do not know what triggers the pathogenic outbreaks of *L. zosterae*, given that the endophyte has been and remains omnipresent in eelgrass beds ([31], pre-wasting disease; [34], post-wasting disease in the 1930s; and [32] contemporary eelgrass beds).

Thus, a method was needed that allows the determination of *L. zosterae* abundance independent of the presence or absence of lesions. To this end, we previously developed a quantitative PCR (QPCR) assay based on species specific ITS primers [35], using DNA extraction from live or dried plant tissue.

In the present study, we (1) surveyed *Z. marina* tissue with our QPCR assay across 19 locations of its European range including Portugal, Germany, Denmark, southern Norway and western Sweden; (2) we compared our assay against the presence of lesions and success in isolating *L. zosterae* (for a subset of five locations) and (3) followed *L. zosterae* concentration over time at one western Baltic and one in the Wadden Sea location. The goal of the study was to establish a baseline of prevalence of the endophyte including temporal variation in infection.

## Materials and Methods

### Sampling

**Figure 1:**
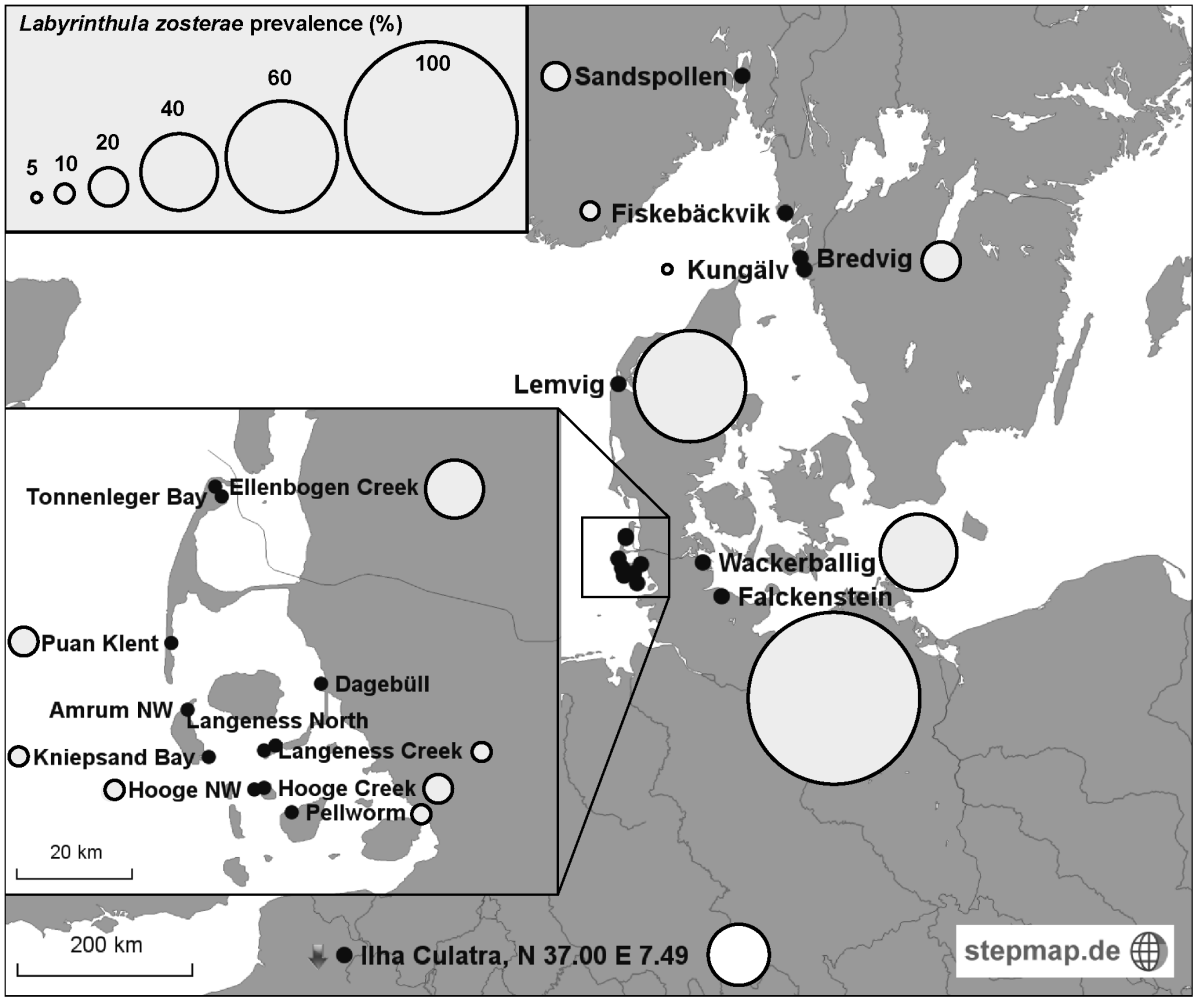
Prevalences of *Labyrinthula zosterae* in *Zostera marina* populations. Circle size proportional to percent prevalence, n = 18–21.

In total, we sampled 19 coastal sited in a water depth of 0.5–3 m (Fig. 1). Eighteen of the 19 sites were situated within the affected region of the 1930’s wasting disease epidemic, while they presently show no signs of decline due to wasting disease (Fig. 1). We were particularly interested to analyze the few remnant permanently submerged *Zostera marina* populations in Wadden Sea tidal creeks, because they are the only subtidal sites that recovered after the wasting disease. These subtidal populations consist of vegetation patches of 0.5–5 m width, distributed along creek banks (33% cover, ±5.5 SE). The intertidal populations sampled in the Wadden Sea are continuous but show sparse eelgrass coverage (mean of all sample sites: 13.4% ±0.5 SE) with low shoot densities (71 shoots m^−2^±1.8 SE). Although intertidal plants are phenotypically distinct from subtidal *Z. marina* (e.g. shoot length_Sylt_intertidal_september_2012_∶24.7 cm ±0.9 SE, shoot length_ Sylt_subtidal_september_2012_∶63.3 cm ±2.9 SE). Microsatellite analysis confirmed low but significant genetic differentiation (*F*_ST_ = 0.009, P = 0.067) between Wadden Sea populations, resulting from divergent selection detected on genes linked with three of 25 microsatellite loci tested [36]. All other populations in this study were continuous eelgrass beds in 0.5–3 m water depth, extending over several 100 of m^2^ (Table 1).

**Table 1:** Sampling locations, salinity and sample size for assessing spatial variance in abundance and prevalence.

Area		Location		Geograph.coordinates		Sampling date		Salinity (psu)		N		% leaves with lesions		% pos. cult. from leaves with lesions	
Langeness, Wadden Sea, Germany		Langeness North		N 54.6396 E 08.5781		22.07.12		>30		20		0		No data	
		Langeness Creek*		N 54.6320 E 08.5440		28.06.11		>30		20		13		No data	
Hooge, Wadden Sea, Germany		Hooge-NW		N 54.5700 E 08.5180		23.07.12		>30		20		0		No data	
		Hooge Creek		N 54.5723 E 08.5240		23.07.12		>30		20		0		No data	
Amrum, Wadden Sea Germany		Kniepsand Bay		N 54.6203 E 08.3970		20.07.12		>30		20		0		No data	
		Amrum-NW		N 54.6960 E 08.3400		19.07.12		>30		20		0		No data	
Pellworm, Wadden Sea, Germany		Pellworm		N 54.5504 E 08.5990		26.07.12		>30		20		0		No data	
Dagebüll, Wadden Sea, Germany		Dagebüll		N 54.7212 E 08.7051		24.07.12		>30		20		0		No data	
Sylt, Wadden Sea, Germany		Puan Klent		N 54.0798 E 08.2960		18.07.12		>30		20		2		No data	
		Tonnenleger Bay		N 55.0258 E 08.4323		17.07.12		>30		20		0		No data	
		Ellenbogen Creek		N 55.0410 E 08.4130		04.07.11		>30		20		40		42.86	
Limfjord, Denmark		Lemvik*		N 56.6300 E 08.2961		28.05.11		>30		19		58		100.00	
Skagerrak, Norway		Sandspollen*		N 59.6657 E 10.5869		10.05.10		20–25		21		57		100.00	
Åbyfjord, Sweden		Fiskebäckvik*		N 58.3362 E 11.4078		01.06.11		20–25		18		No data		11.11	
Gullmarsfjord, Sweden	Bredvik+Snäckebacke-bukten		N 58.1987 E 11.3244		03.07.11		20–30		20		No data		No data		
	Kungälv		N 57.5405 E 11.4083		04.07.11		6–14		20		No data		No data		
Flensburg Fjord, Germany	Wackerballig*		N 54.7557 E 09.8668		12.07.11		15–17		19		80		100.00		
Kiel Fjord, Germany	Falckenstein		N 54.3954 E 10.1935		15.07.11		15–17		20		95		100.00		
Faro lagoon, Portugal	Ilha Culatra		N 37.0005 W 07.4921		05.08.11		36		20		No data		No data		

The percentage of *Zostera marina* plants with lesions and successful *Labyrinthula* isolations are shown where available,

* = subset of 5 populations chosen for methods comparison.

At each site, fresh leaves of at least twenty *Z. marina*-shoots were collected between May and August of the years 2010 (1 site), 2011 (8 sites) and 2012 (10 sites, Table 1), separately stored in Zip-lock bags with ambient sea water and kept cool until return to the lab 1–3 days later. Sampling at Ellenbogen Creek was permitted by nature conservation authority and Mr. Diedrichsen, the owner of this private property. We took care that by picking a leaf piece the entire plant was kept alive in situ and/or sampled outside areas not open to public. Therefore no special permission was necessary at all other sites.

Before starting the spatial survey, we wanted to address within-plant variation in *Labyrinthula zosterae* abundance. To this end DNA was extracted from all leaves of eight individual plants of two sites (Lemvig and Wackerballig), dividing each leaf in three sections (top, middle, basis). Initial QPCR-assay results revealed that the highest *L. zosterae* prevalences and/or abundances were found in the middle part of the 3^rd^ oldest leaf (for means and statistical tests see Tables 2 and 3); therefore, we analyzed the 3^rd^ leave in all subsequent samples.

**Table 2:** Mean *Labyrinthula zosterae* abundance and prevalence in different leaf parts.

Leaf part	N	*L. zosterae* cells×mgplant DW^−1^	Std. Err	Prevalence (%)
Top	27	0.30	0.14	18.92
Middle	22	71.31	67.54	38.71
Basis	19	9.37	3.08	31.03

Abundance per g *Zostera marina* dry weight (DW) with standard errors: Wilcoxon-Kruskal-Wallis Test_leaf part_: df = 2, X^2^ = 6.05, p = 0.05, planned comparison_abundance_: top< middle **, basis<middle**. Prevalence (%): Nominal logistic regression_leaf parts_: df = 2, deviance = 14.47, p = 0.001, planned comparison_prevalence_: top< middle*, * = significantly different at p>0.05, ** = p<0.02.

**Table 3:** Mean *Labyrinthula zosterae* abundance and prevalence among different *Zostera marina* leaves.

Leaf number	N	*L. zosterae* cells×mg plant DW^−1^	Std. Err.	*Prevalence (%)*
1	16	6.00	2.52	*12.50*
2	19	5.22	2.01	*10.53*
3	18	6.00	80.84	*50.00*
4	12	0.33	0.09	*33.33*
5	3	2.48	0.00	*One data point only*

Abundance per g *Zostera marina* weight (DW) with standard errors: Wilcoxon-Kruskal-Wallis-test_leaf number_: df = 4, X^2^ = 5.37, p = 0.25). Prevalence (%): Nominal logistic regression_leaf number_: df = 4, deviance = 9.71, p = 0.05, planned comparison_prevalence_: leaf 2<3**, ** = significantly different at p>0.02.

After sampling, leaves from all populations were air dried. Leaves from five of these populations (Table 1) were additionally examined for black lesions on the leaves. Then all leaves were cut in half, longitudinally. One half was dried for later DNA extraction, the other half served as inoculum for cultivation of *Labyrinthula zosterae* on seawater-agar medium.

To assess temporal variation in *L. zosterae* prevalence and abundance, the same population was sampled 14x in Falckenstein (7.4., 21.4., 5.5., 19.5., 9.6., 23.6., 7.7., 15.7., 5.8., 28.9., 1.11. and 28.1.2011, 23.2. and 25.3.2012) and 6x at Ellenbogen creek (18.5., 9.6., 4.7., 4.8., 5.9. und 10.11.2011).

### DNA Extraction

Ca. 2–4 mg of dried leaf material was first ground in a ball mill (Retsch, Germany) at maximal speed setting for 5 min. DNA extractions of *L. zosterae* were performed with an Invisorb spin tissue mini kit (Invitek, Berlin, Germany) following the manufacturer’s instructions. To enhance extraction efficiency and to ensure that even low amounts of target DNA were carried through the filter absorption steps, 1 µL (containing ∼500 ng) of UltraPure™ salmon sperm DNA solution (Invitrogen, life technologies, USA) was added to each extraction to saturate silica columns with DNA. Target DNA was purified using a one-step PCR inhibitor removal kit (Zymo Research, USA).

### Quantitative PCR (QPCR)-assay

Following on the original assay protocol of Bergmann [35] we modified the method to enhance specificity and sensitivity by developing a novel, TaqMan based assay with the consensus sequence of *Labyrinthula zosterae* ([35]; accession numbers JN121409-13). Using the software PrimerXpress (Applied Biosystems) the forward primer Laby_ITS_Taq_f: TTGAACGTAACATTCGACTTTCGT and the reverse primer Laby_ITS_Taq_r: ACGCATGAAGCGGTCTTCTT were identified, along with the probe Laby_ITS_Taq_pr: TGGACGAGTGTGTTTTG that carried the fluorescence label 6-Fam at the 5′ end and the dark quencher BHQ-1 at the 3′ end. Reactions were carried out using standard conditions recommended by the manufacturer using the 10 µL TaqMan universal Master Mix (Applied Biosystems, now Life Technologies) in a 20 µL reaction volume: 2 µL 1∶10 diluted template DNA, 2.4 µL (40.8 nM) of the two primers, 2.4 µL Milli-Q H_2_O and 0.8 µL probe (50 nM), respectively. The thermo-cycling program on a Step-One QPCR machine was 2 min at 50°C and 10 min at 95°C, followed by 48 cycles at 95°C for 15 s and 1 min at 60°C. All samples were tested in triplicate and the standard deviation of triplicates never exceeded 0.3 units of cycle threshold (Ct). Only CT values <39 were considered. Standard curves using preparations of *Labyrinthula zosterae* with known cell numbers attained correlation coefficients between r^2^ = 0.97 and 0.99 and a detection limit of ∼0.01 cells. Abundance as the number of *L. zosterae* cells in each milligram (dry weight) *Zostera marina* sample was calculated from the linear regression of the standard curve (standard cell number against mean standard Ct calculated from all QPCR reactions; 150 cells 22.493 Ct ±0.060 SE, 15 cells = 27.080 Ct ±0.080 SE, 0.5cells = 32.215 Ct ±0.125 SE).
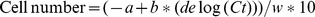
where a = intercept, b = slope and w = sample dry weight. Cell numbers were multiplied by 10 because the samples were diluted 1∶10 prior QPCR.

Prevalence was calculated as the percentage of samples of each site with a Ct<39.

### Cultures

#### Seawater-agar medium

For one liter of seawater-agar medium (for 50 Petri-dishes 10 cm in diameter) : 12 g agar (bacteriological grade, Roth, Germany ), 1 g glucose, 0.1 g yeast extract (Roth, Germany), 0.1 g peptone (Fluka, Germany) in 1 L Milli-Q water were mixed and autoclaved 30 min at 121°C. Immediately following the autoclave step, 25 g Instant Ocean (Instant Ocean, Spectrum Brands, USA) artificial sea salt was added (salinity: 25 psu). After cooling to 50°C, 25 mL Penicillin-Streptomycin (MP Biomedicals, USA) and 10 mL horse serum (Invitrogen, USA) were added, mixed, and the medium poured immediately.

#### Labyrinthula-isolation

Ca. 2 cm-long leaf pieces taken from the middle part of each 3^rd^ leaf were dipped in 0.5% hypochlorite (bleach) solution in seawater for 20 s of surface sterilization, rinsed with Milli-Q water for 10 s and soaked in artificial seawater for 1 min. Washed leaf samples were separately placed on the agar plates and incubated at 25°C in a climate cabinet without light. Cultures were checked under the dissecting scope after three, five and eight days for growing *L. zosterae*.

### Statistical Analysis

To compare mean *Labyrinthula zosterae*-abundances (cell numbers obtained by QPCR-assay) we used non-parametric tests because data were markedly non-normally distributed. *L. zosterae* abundance in different positions of the leaf/in leaves of different age was compared by Wilcoxon/Kruskal-Wallis-tests (implemented in software JMP 9, SAS Institute, USA) followed by planned contrasts to identify which leaf parts/leaves were different. Likewise, spatial and temporal patterns in *L. zosterae* abundance were tested for statistically significant variation using Wilcoxon/Kruskal-Wallis-tests (implemented in software JMP 9, SAS Institute, USA). Nominal logistic regression was applied to nominal data, i.g. the presence/absence of lesions on leaves and prevalence measurements. Prevalence was defined depending on the method used. Using culturing, it was defined as successful/unsuccessful isolation of *L. zosterae* from fresh leaf material. When using QPCR on dried leaf material, positive prevalence was defined as a PCR reaction with a Ct-value <39, from dried leaf material.

## Results

### Prevalence and Abundance of *Labyrinthula Zosterae*

Using the QPCR assay, *L. zosterae* was present in 16 of 19 populations tested, with a statistically significant variation of prevalence among sites (Nominal logistic regression_site_: df = 18, deviance = 116.06, p = 0.0001). Because we had no *a priori* expectations about site-specific abundances, we did not perform any post-hoc tests. The highest prevalence of 88.9% was found in Falckenstein, the population in Kiel Fjord. Lemvig plants were ranked second in terms of prevalence (58%, Fig. 1). The Swedish Kungälv population showed the lowest prevalence (5%). No *L. zosterae* was found in Tonnenleger Bay, Amrum NW, Dagebüll and Langeness North (intertidal populations, Table 1).

**Figure 2:**
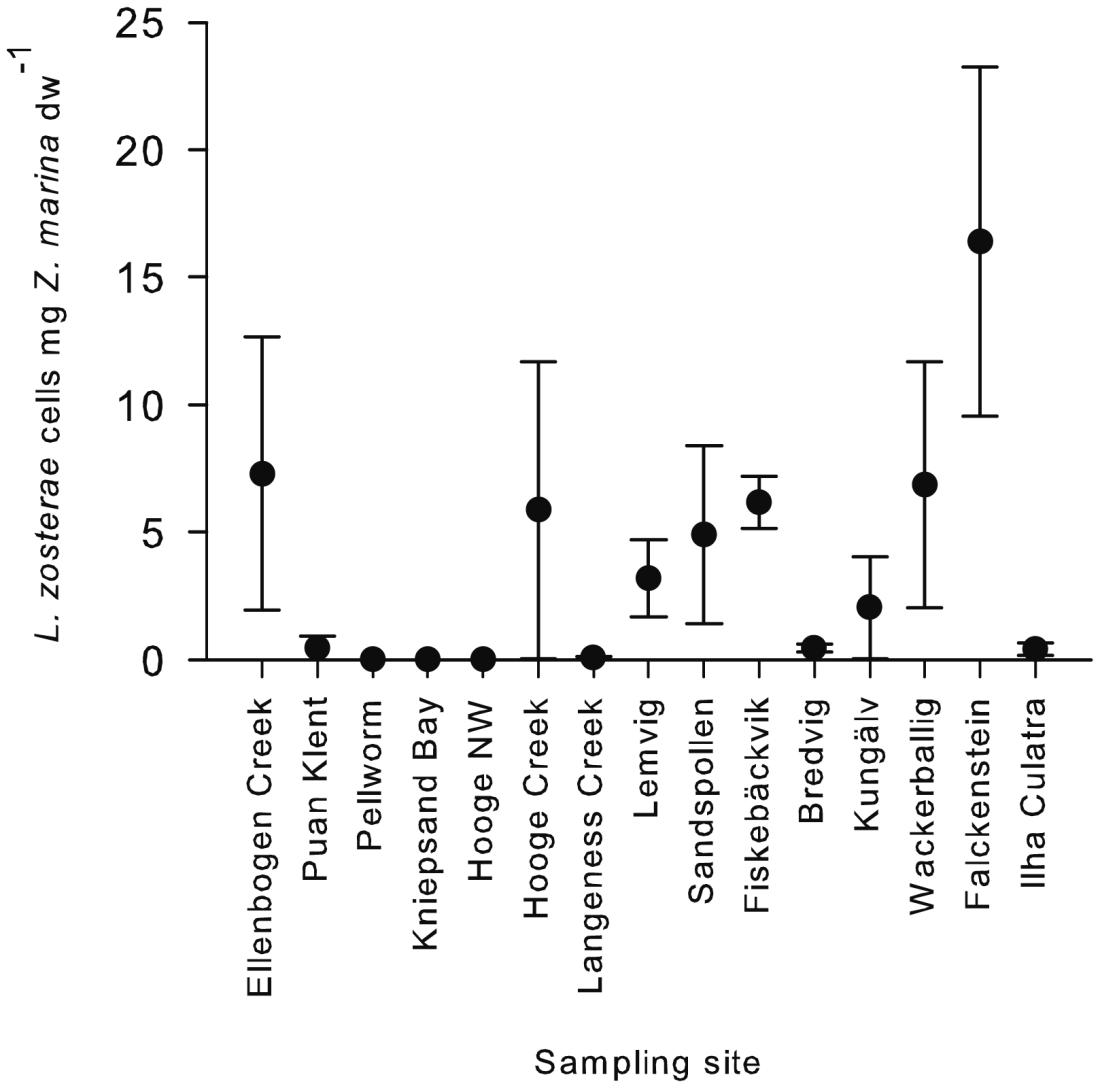
Differences in the abundance of *Labyrinthula zosterae* in infected *Zostera marina* plants from 15 sites. Means with standard error bars, N = 18–21.

**Figure 3:**
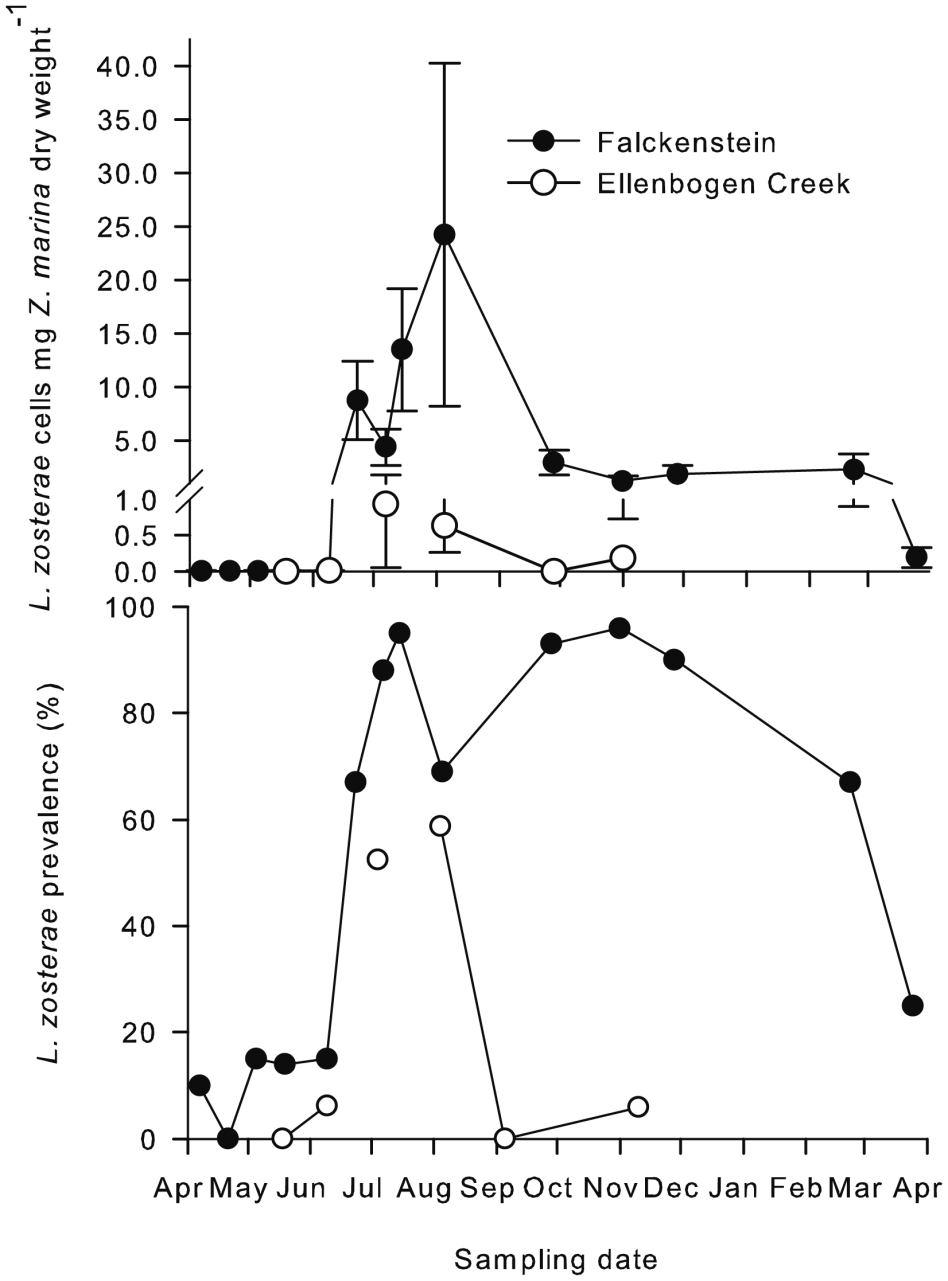
Temporal variation in the abundance and prevalence of *Labyrinthula zosterae* in infected *Zostera marina* plants. Means with standard error bars, N = 10–25, Falckenstein = Baltic Sea, Ellenbogen Creek = Wadden Sea (sublitoral).

The abundance of *L. zosterae* was standardized relative to eelgrass dry weight (DW) and revealed high variation within and among sites (minimum: 0.01 *L. zosterae* cells mg^−1^ plant DW, maximum: 504 *L. zosterae* cells mg^−1^ plant DW, Fig. 2). Note that cell numbers <1 are possible because the amplified ITS-region belongs to the multi-copy rDNA gene and the detection limit per PCR-reaction was 0.01 cells. Similar to prevalence, abundance was highest in Falckenstein (16.40 cells mg^−1^ plant DW ±6.84 SE), followed by Fiskebäckvik (6.17 cells mg^−1^ plant DW ±1.03 SE) as shown in Fig. 3. The lowest abundances were found in the positive samples from Hooge NW and Pellworm Creek (0.01 cells mg^−1^ plant DW). Site differences were significant (Wilkoxon/Kruskal-Wallis-test_site_: χ^2^ = 25.27, df = 14, p = 0.032; note that only positive values were included into the analysis resulting in an exclusion of sites without *L. zosterae*).

### Lesion, Isolation and Prevalence of *Labyrinthula Zosterae*

**Figure 4:**
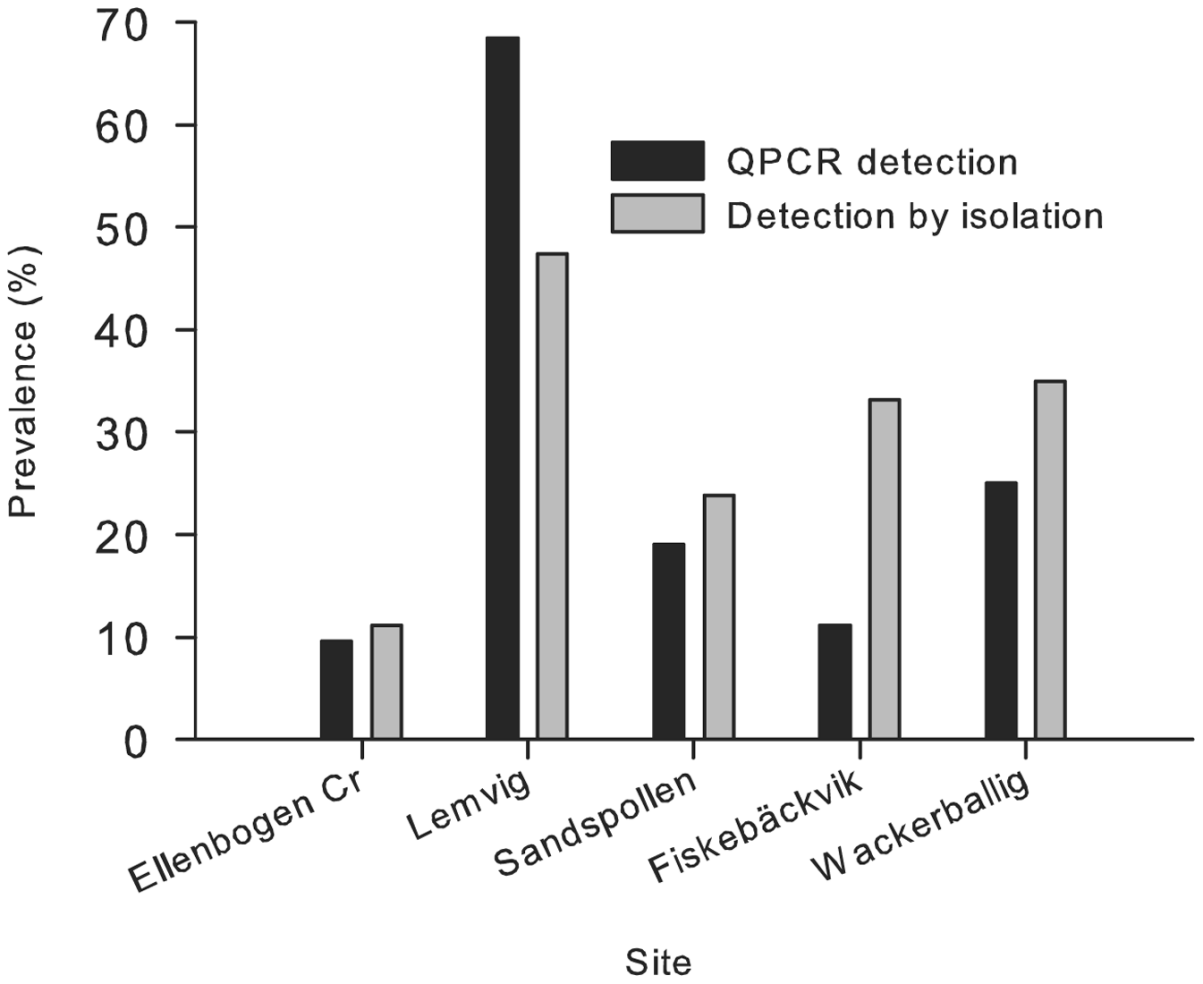
Differences in the prevalence of *Labyrinthula zosterae* in *Zostera marina*-samples detected by QPCR-assay versus isolation. Samples from five sites, isolation on seawater-agar-culture plates, N = 18–21.

For a subset of five sites, we investigated the presence of lesions and the isolation success of *Labyrinthula zosterae* in addition to QPCR-assay analysis. Prevalences of *Labyrinthula zosterae* assessed as isolation success via cultivation did not differ significantly from obtained via the QPCR assay. We analyzed the method applied together with site differences in prevalence in one model. Differences were only found for site and not for the method used (Fig. 4, Nominal logistic regression_method and site_: method: df = 1, deviance = 0.04, p = 0. 850, site: df = 2, deviance = 20.28, p = 0.0004, method×site: df: 4, deviance 3.245, p = 0.5177, ns). The mean prevalence across all sites was 26% for the QPCR-approach and 30% for the isolation approach.

The percentage of leaves with lesions (small black or brown spots, between 1 mm and 2 cm in diameter) differed markedly among populations ranging from 11% in Fiskebäckvik to 80% in Wackerballig (Table 1, Nominal logistic regression_lesion_ for site: df = 4, deviance = 17.81, p = 0.0013). Across all sites, the probability of obtaining a positive *L. zosterae* culture or a positive QPCR result was significantly higher in leaves with lesions that without, although the protist was also found in plants without lesions. 48.8% of the leaves where *L. zosterae* has been detected by QPCR showed lesions, whereas the protist was only found in 10.4% leaves without lesions (Nominal logistic regression_lesion_: df = 1, deviance = 15.87, p = 0.001, log odds ratio = 1.39, SE = 0.585). Using isolation, *L. zosterae* could be detected in 56.5% leaves with lesions but only in 8.3% without (Nominal logistic regression_lesion:_ df = 1, deviance = 32.37, p = 0.0001, log odds ratio = 2.88, SE = 0.616).

Interestingly, isolates of *L. zosterae* were easily obtained from lesions on the leaves at Sandspollen, Fiskebäckvik, Wackerballig and Lemvig, whereas this was not the case with the leaves from sublitoral eelgrass plants in Ellenbogen Creek. Here, 57% of the *Labyrinthula* isolated came from green leaves without any lesions.

### Temporal Variation in Abundance and Prevalence of *Labyrinthula zosterae*

At two selected sites, prevalence and abundance of *L. zosterae* were monitored throughout one year. Overall the temporal patterns were congruent. Prevalence data varied strongly and ranged between 0 and 25% between April and June, 67–95% between the end of June and September. At the western Baltic Sea site of Falckenstein (Table 1 and Fig. 3) *L. zosterae* occurred at very low abundances between April and June (0.01–0.09 cells mg^−1^ *Z. marina* dry weight), increasing from the end of June and September (4.4–24.3 cells mg^−1^ *Z. marina* dry weight) and declining from October until March (ca. 1 cell/mg *Z. marina* dry weight (Wilkoxon/Kruskal-Wallis-test_sampling date_: df = 12, χ^2^ = 141.40, p<0.0001). The Wadden Sea site at Ellenbogen Creek (Table 1 and Fig. 3) revealed much lower prevalences and abundances than the Baltic Sea Falckenstein location. Here, only about 20% of plants were infected during the July-August period and abundances also remained low (0.6–0.9 cells mg^−1^ *Z. marina* dry weight, Wilkoxon/Kruskal-Wallis-test_sampling date_: df = 4, χ^2^ = 28.256, p<0.0001).

## Discussion

After nearly a century of investigations on *Labyrinthula zosterae* as putative agent of eelgrass wasting disease there is still no conclusive picture of what triggers pathogenic outbreaks. We show here that background prevalence is extremely high in contemporary eelgrass beds in northern Europe, with up to 89% of the plants carrying *L. zosterae*. Using a specific QPCR assay, we show that *Labyrinthula zosterae* is present in almost all populations assessed even though most plants showed few lesions, let alone signs of an epidemic outbreak. The QPCR assay thus provides a valuable tool to assess background levels (∼0.01 cells mg^−1^ DW) of *L. zosterae* independent of lesions. Prevalence, as determined by either QPCR data or isolation and culture were comparable. Since the latter is far more laborious and slow, preference should be given to a QPCR assay which also works with dried samples. A direct comparison of QPCR values with the “wasting disease index” [33] has to be interpreted with caution, as the QPCR and the wasting index measure different processes. The wasting disease index reflects the cumulative pathogenic effects of a *L. zosterae* infection (including e.g. defense reactions of the plant), whereas the QPCR value reflects abundance only. The two should be seen as complementary.

Currently we do not know whether the very low background concentrations of the *L. zosterae* endophyte in winter and spring are to the only inoculum that gives rise to high abundances during summer, or whether eelgrass leaves are secondarily infected every year from *L. zosterae* spores the environment. Although a number of life history studies on *L. zosterae* have been conducted earlier [37]–[40], the details of zoospore formation as well as the emergence and location of resting stages (cysts) in the environment remain unknown. While have not yet searched for resting stages in the sediments and/or water column our QPCR approach may be the suitable tool to do so. Equally unknown is how the endophyte disperses which could take place via the drift of decaying infected leaves. *L. zosterae* can be transmitted rapidly by direct contact of leaves (AC Bockelmann, personal observation).

With a mean value of 5.7 *L. zosterae* cells mg^−1^ *Z. marina* dry weight (±1.9 SE), abundances of *L. zosterae* seem low on an absolute scale but are consistent with a scenario of chronic, non-pathogenic infection, while the variation across and among sites is very high. *Z. marina* plants from four intertidal sites in the Wadden Sea were completely uninfected, even in summer. High intra- as well as inter-population variability may be due to stochastic infection dynamics [41], [42], genotypic resistance effects of the host, as shown for other pathogen-host associations [43], [44] or due to differential physiological activity among leaves and among individuals. For example, a single eelgrass shoot from one individual can harbor 20,000 times as many *L. zosterae* cells as a shoot from another individual just a few meters away (this study). Rapid changes in abundance of *Labyrinthula* spp. have been shown in culture where cells can spread 10 mm hr^−1^ [30]; spread has also been shown to correlate with reduced photosynthetic capacity across an infected area of the leaf at a velocity of 0.8 mm hr^−1^ [45]. Thus infection of the physiologically most active parts of the plants undoubtedly contributes to high intra-individual variation. The extremely low abundance of *L. zosterae* in subtidal Wadden Sea populations may be a result of high resistance to infection, resulting from the 1930s epidemic which destroyed almost all subtidal eelgrass beds.

Experimental investigations of *L. zosterae* and lesion development revealed that neither high temperatures, nor high salinity or low light availability could be identified as variables that satisfactory explain the 1930’s pandemic [5], [33], [46]–[48]. Next to environmental factors, interactions with biotic effects such as herbivory [49] and competition with epiphytes and bacteria on the leaf surface [50] are likely to impact infection dynamics. Our QPCR assay also provides the opportunity to study historical museum material (AC Bockelmann unpublished) in order to determine whether the *L. zosterae* present in today eelgrass meadows is the same strain that caused the 1930’s wasting disease epidemic and thus provide a clue about the endophyte’s possible origins.

A commensalistic or even mutualistic relationship [43], [44] for *Labyrinthula* species is also worthy of further investigation, as has been shown for many terrestrial plant-endophyte associations [43], [44], [51]–[53]. Several other *Labyrinthula* species have now been identified in the Baltic [32], suggesting that a commensally association may be more likely than previously supposed. It is conceivable that the presence of the endophyte in low concentration confers some sort of chemical protection against other infections like known from bacteria or fungi [53], [54]. Schmoller [55] found that in culture *Labyrinthula coenocystis* can actually be nourished by a bacterial film. Furthermore, the rapid decay and mineralization of senescent leaves [50] could alleviate nutrient limitation for eelgrass plants. Switches between pathogenic and mutualistic relationships are common in plant-endophyte symbiosis [56], [57], which could also be the case here. There is thus a pressing need to experimentally disentangle the role of different environmental and biotic factors as well as the mechanism of host defense [58].

In culture, morphological differences in colony growth form, cell morphology, and in pathogenicity and infectiousness have been observed, which suggests different genetic backgrounds [59], [60], (AC Bockelmann, personal observation). However, there is currently no genetic or definitive experimental data available. Whereas species differences have been documented using 18S ribosomal rDNA sequence analysis [32], there are currently no genetic markers to distinguish among specific strains that are of commensalistic vs. pathogenic nature.

With climate change resulting in a multitude of altered environmental conditions, for example warmer temperatures and ocean acidification, marine diseases in several taxonomic groups are already noticeably increasing [6], [61]–[63]. Given that endophytes such as *Labyrinthula* species are diverse and that only very few have been studied thus far (as *L. zosterae* for *Z. marina*), it may be useful to other endophytes in addition to *Labyrinthula zosterae* in future studies on eelgrass health and performance [64].
